# Role of NPR1 and KYP in long-lasting induced resistance by β-aminobutyric acid

**DOI:** 10.3389/fpls.2014.00184

**Published:** 2014-05-08

**Authors:** Estrella Luna, Ana López, Jaap Kooiman, Jurriaan Ton

**Affiliations:** ^1^Department of Animal and Plant Sciences, The University of SheffieldSheffield, UK; ^2^Department of Biology, Utrecht UniversityUtrecht, Netherlands

**Keywords:** priming, induced defense, *Arabidopsis*, NPR1, KYP

## Abstract

Priming of defense increases the responsiveness of the plant immune system and can provide broad-spectrum protection against disease. Recent evidence suggests that priming of defense can be inherited epigenetically to following generations. However, the mechanisms of long-lasting defense priming within one generation remains poorly understood. Here, we have investigated the mechanistic basis of long-lasting induced resistance after treatment with β -aminobutyric acid (BABA), an agent that mimics biologically induced resistance phenomena. BABA-induced resistance (BABA-IR) is based on priming of salicylic acid (SA)-dependent and SA-independent defenses. BABA-IR could be detected up to 28 days after treatment of wild-type *Arabidopsis.* This long-lasting component of the induced resistance response requires the regulatory protein NPR1 and is associated with priming of SA-inducible genes. In contrast, NPR1-independent resistance by BABA was transient and had disappeared by 14 days after treatment. Chromatin immunoprecipitation (ChIP) assays revealed no increased acetylation of histone H3K9 at promoters regions of priming-responsive genes, indicating that this post-translational histone modification is not critical for long-term transcriptional priming. Interestingly, the *kyp-6* mutant, which is affected in methyltransferase activity of H3K9, was blocked in long-lasting BABA-IR, indicating a critical requirement of this post-translational histone modification in long-lasting BABA-IR. Considering that KYP suppresses gene transcription through methylation of H3K9 and CpHpG DNA methylation, we propose that KYP enables long-term defense gene priming by silencing suppressor genes of SA/NPR1-dependent genes.

## INTRODUCTION

Plants can resist pathogen attack by increasing the responsiveness of their immune system. This phenomenon typically occurs after perception of stress-indicating signals and is known as priming of defense. Priming provides non-specific protection against a wide range of biotic and abiotic stresses (Conrath et al., 2006; Pastor et al., 2012; Tanou et al., 2012), which is associated with relatively minor costs on growth and reproduction (Van Hulten et al., 2006). Induction of defense priming results in a faster and stronger expression of basal immune responses upon pathogen attack (Conrath et al., 2006, 2011), and can render plants immune if the augmented defense reaction precedes immune-suppression by pathogen (Ahmad et al., 2010). In most cases, however, defense priming slows down pathogen colonization and reduces disease. Research over the past decades has identified various chemicals that can mimic biologically induced priming responses. These chemicals are often plant-derived signaling metabolites, such as salicylic acid (SA; Shirasu et al., 1997), jasmonic acid (JA; Kauss et al., 1994; Conrath et al., 2002), azelaic acid (Jung et al., 2009), or herbivore-induced volatiles (Ton et al., 2007; Frost et al., 2008). There are also xenobiotic chemicals that can trigger defense priming in plants. Amongst these, the non-protein amino acid β-aminobutyric acid (BABA; Zimmerli et al., 2000; Ton et al., 2005) and benzo-thiadiazole-7-carbothioic acid *S*-methyl ester (BTH; Kohler et al., 2002) have emerged as popular agents to study the mechanistic basis of defense priming in plants (Conrath, 2011).

BABA-induced resistance (BABA-IR) mimics component of defense priming that are active during pathogen-induced systemic acquired resistance (SAR) and rhizobacteria-induced systemic resistance (ISR; Van der Ent et al., 2009). Consequently, it provides protection against an exceptionally broad range of pathogens and insects. The signaling pathways controlling BABA-IR against the bacterial pathogen *Pseudomonas syringae* pv *tomato* DC3000 relies on production of the plant hormone salicylic acid (SA) and a functional non-expressor of PR GENES (NPR1) protein (Zimmerli et al., 2000). However, BABA-IR against the oomycete pathogen *Hyaloperonospora arabidopsidis* and the necrotrophic fungi *Alternaria brassicicola* and *Plectosphaerella cucumerina* can function independently from NPR1, but requires components of the abcisic acid (ABA) signaling pathway (Ton and Mauch-Mani, 2004; Ton et al., 2005). Both pathways operate independently from each other and provide different mechanisms of defense priming (Ton et al., 2005). The NPR1-independent pathway primes cell wall defense, which leads to augmented deposition of callose-rich papillae after pathogen attack. On the other hand, the NPR1-dependent pathway controls priming of SA-dependent genes, which is marked by enhanced transcription of NPR1-dependent transcription factor (TF) genes that control SA-dependent gene induction (Van der Ent et al., 2009). The latter finding suggested that greater abundance of defense regulatory TFs contributes to transcriptional priming of SA-inducible defense genes. However, TFs have limited turn-over times and their enhanced accumulation after application of a single priming stimulus is not a satisfactory explanation for a long-lasting induced resistance response.

Epigenetic mechanisms, such as histone modifications or DNA methylation, have emerged as important regulatory mechanisms in plant immunity (Alvarez et al., 2010). There is ample evidence that post-translational modifications of histone proteins are influences by JA-, SA-, and ABA-dependent signaling pathways (Devoto et al., 2002; Mosher et al., 2006; Walley et al., 2008; Cho et al., 2012). Furthermore, exposure to disease, herbivores and abiotic stresses can have profound impacts on patterns of symmetric and asymmetric DNA methylation (Pavet et al., 2006; Boyko et al., 2010; Verhoeven et al., 2010; Dowen et al., 2012). It is, therefore, not surprising that priming of defense has been associated with epigenetic regulatory mechanisms (Conrath, 2011; Pastor et al., 2012). First evidence for an epigenetic basis of defense priming came from Jaskiewicz et al. (2011), who demonstrated that infection of *Arabidopsis* by *Pseudomonas syringae* pv. *maculicola* primes stress-inducible expression of transcription factor genes via NPR1-dependent modifications of histone H3 at their promoter regions. Furthermore, López et al. (2011) demonstrated that mutants blocked in RNA-directed DNA methylation are primed to activate SA-inducible defense genes, which was associated with H3 modifications marking a facilitated state of gene transcription: acetylation at lysine residue 9 (H3K9ac) and triple-methylation at lysine 4. Hence, defense priming is often associated with post-translational histone modifications at promoter regions of primed defense genes.

Recently, three independent research groups provided evidence that priming of defense can be inherited epigenetically from isogenic plants that had been treated with pathogens, herbivores, or BABA (Luna et al., 2012; Rasmann et al., 2012; Slaughter et al., 2012). Although these studies demonstrated an epigenetic component of defense priming, the extent by which epigenetic regulation contributes to long-lasting defense priming within one plant generation remains unknown. In this study, we have investigated the mechanisms controlling durable maintenance of defense priming in individual plants after treatment with the chemical agent BABA. We show that only the NPR1-dependent component of BABA-IR is long-lasting in *Arabidopsis*, which is associated with priming of SA-inducible defense genes. Furthermore, we provide evidence that this long-lasting defense resistance requires the histone methyltransferase KYP.

## MATERIALS AND METHODS

### PLANT MATERIAL, GROWTH CONDITIONS, AND EXPERIMENTAL DESIGN

**FIGURE 1:**
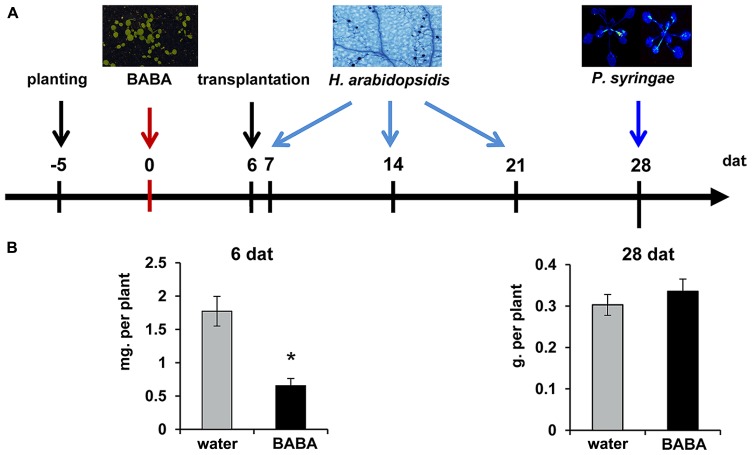
**Experimental design to determine durability of induced resistance**. **(A)** Five-day-old seedlings were soil-drenched with water or BABA (40 mg/L) and transplanted to BABA-free soil at 6 days after induction treatment (dat). Plants were examined for shoot fresh weight at 6 and 28 days after treatment (dat), or inoculated with either *Hyaloperonospora arabidopsidis* WACO9 (7, 14, 21 dat), or *Pseudomonas syringae* pv. *tomato* DC3000 (*Pst*-luxCDABE; 28 dat). **(B)** Mean shoot fresh weights of water- and BABA-treated plants at 6 and 28 dat. Error bars represent the SEM. Asterisk indicates a statistically significant difference between water- and BABA-treated plants (Student’s *t*-test; α < 0.05; *n* = 15).

*Arabidopsis thaliana* (Col-0), *npr1-1* (Despres et al., 2003), and *kyp-6* (Alonso et al., 2003; Chan et al., 2006) were cultivated in a growth chamber with a 8-h day (150 μE m^-2^ s^-1^ at 20°C) and 16-h night (18°C) under at 65% relative humidity. Seeds were planted in 60-ml pots containing a 50% (v/v) sand/M3 mixture and kept at 4°C in the dark for 2 days to break dormancy. Five day-old seedlings were soil-drenched with water or BABA solution (Sigma-Aldrich; Cat.: A4, 420–7) to a final concentration of 40 mg/L in the soil. Six days after treatment, seedlings were transplanted to BABA-free soil (**Figure 1A**). At different time-points after treatment, plants were examined for differences in fresh weight (7 and 28 days), inoculated with *H. arabidopsidis* (7, 14, and 21 days), inoculated with *Pseudomonas syringae* pv. *tomato* DC3000 *luxCDABE* (*Pst*-luxCDABE; Fan et al., 2008; 28 days), or examined for priming of salicylic acid (SA)-inducible gene expression and chromatin modification (28 days).

### GROWTH ANALYSIS AND INDUCED RESISTANCE BIOASSAYS

Plant growth was analyzed by measuring shoot fresh weights at 6 and 28 days after induction treatment (seedlings were excised from the roots, including hypocotyls). Inoculation and determination of pathogen colonization were performed as described previously (Luna et al., 2012). Colonization by *H. arabidopsidis* was scored at 6 days after inoculation. Colonization by *Pst*-luxCDABE (Fan et al., 2008) was quantified at 3 days after inoculation.

### GENE EXPRESSION ANALYSIS

Water- and BABA-treated plants (*n* = 30) were sprayed with 0.5 mM SA (Sodium salicylate; Sigma-Aldrich; Cat.: S3007). At 0, 4, 8, and 24 h after application of SA, at least three biologically replicated samples containing pooled material from individual pots, were snap frozen in liquid nitrogen. RNA extraction, cDNA synthesis, and reverse-transcriptase quantitative PCR (RT-qPCR) with gene-specific primers were entirely performed as described before (Luna et al., 2012). Fold induction values were normalized to average 2^ΔCt^ values relative to time-point 0 h before SA application of control-treated plants.

### CHROMATIN IMMUNOPRECIPITATION

Chromatin immunoprecipitation (ChIP) assays were carried out as described in the manufacturer’s protocol (EpiQuik Plant ChIP kit; Epigentek, Brooklyn, NY, USA), using mature leaves from 5-week-old plants. For each experiment, at least three biologically replicated samples were collected, each consisting of rosettes from five to seven plants. Chromatin samples were immunoprecipitated using antibodies against acetyl-histone H3K9 (Millipore 07-352). Before and after immunoprecipitation, DNA abundance in chromatin extracts was analyzed by quantitative PCR, using the ABI PRISM^®^ 7900 HT sequence detection system. Two technically replicated reactions per sample were performed in a final reaction volume of 25 μl, containing Jump Start SYBR Green (Sigma-S4438). Sequence-specific primers were used to amplify promoter DNA from *PR1*, *WRKY6*, *WRKY29*, *WRKY53* (Jaskiewicz et al., 2011), and *WRKY70* (Fw: AATTAGATTCAAGTCCACAACCAA Rv: ATCAAGAAATTGTCATCCAACAC). Results were normalized to DNA amounts in the input control, as described by Haring et al. (2007) with modifications. To prevent possible bias to inaccurate estimations of input DNA, two independent DNA extractions were performed from each chromatin extract. Only if input values differed less than 0.25 *C*_t_ values, samples were considered reliable for further analysis and *C*_t_ input values were averaged for normalization of immunoprecipitated DNA.

### STATISTICS

Average shoot fresh weights and % bacterial bioluminescence were based on at least 15 individual plants per treatment and were analyzed for statistical differences by Student’s *t*-tests (α = 0.05; SPSS, v19.0). *H. arabidopsidis* class distributions were based on 50–100 leaves and differences between treatments were analyzed for statistical significance by χ2 contingency tests using SPSS, v19.0. Average fold-change values of gene expression and H3K9ac levels were based on three biological replicates per treatment and statistical differences were determined by Student’s *t*-tests (α = 0.05; SPSS, v19.0). Each experiment was repeated twice from the onset.

## RESULTS

### NO LONG-LASTING IMPACTS OF BABA ON PLANT DEVELOPMENT

Induction of disease resistance by BABA can reduce growth of *Arabidopsis* (Van Hulten et al., 2006; Wu et al., 2010). To examine the long-lasting impact of BABA on plant development, 5-day-old seedlings were soil-drenched with 40 mg/L BABA. Seedlings were kept in BABA-drenched soil for 6 days, after which they were transferred to un-treated soil in order to exclude ongoing induction by excess amounts of soil-based BABA (**Figure 1A**). Fresh weight analysis of shoots at 6 days after treatment revealed a statistically significant reduction of plant growth in BABA-treated plants (**Figure 1B**). However, no growth differences were apparent by 28 days after treatment, indicating that BABA-treated plants can fully recover from the induction treatment.

### INDUCED RESISTANCE BY BABA LASTS UP TO 4 WEEKS AFTER TREATMENT

**FIGURE 2:**
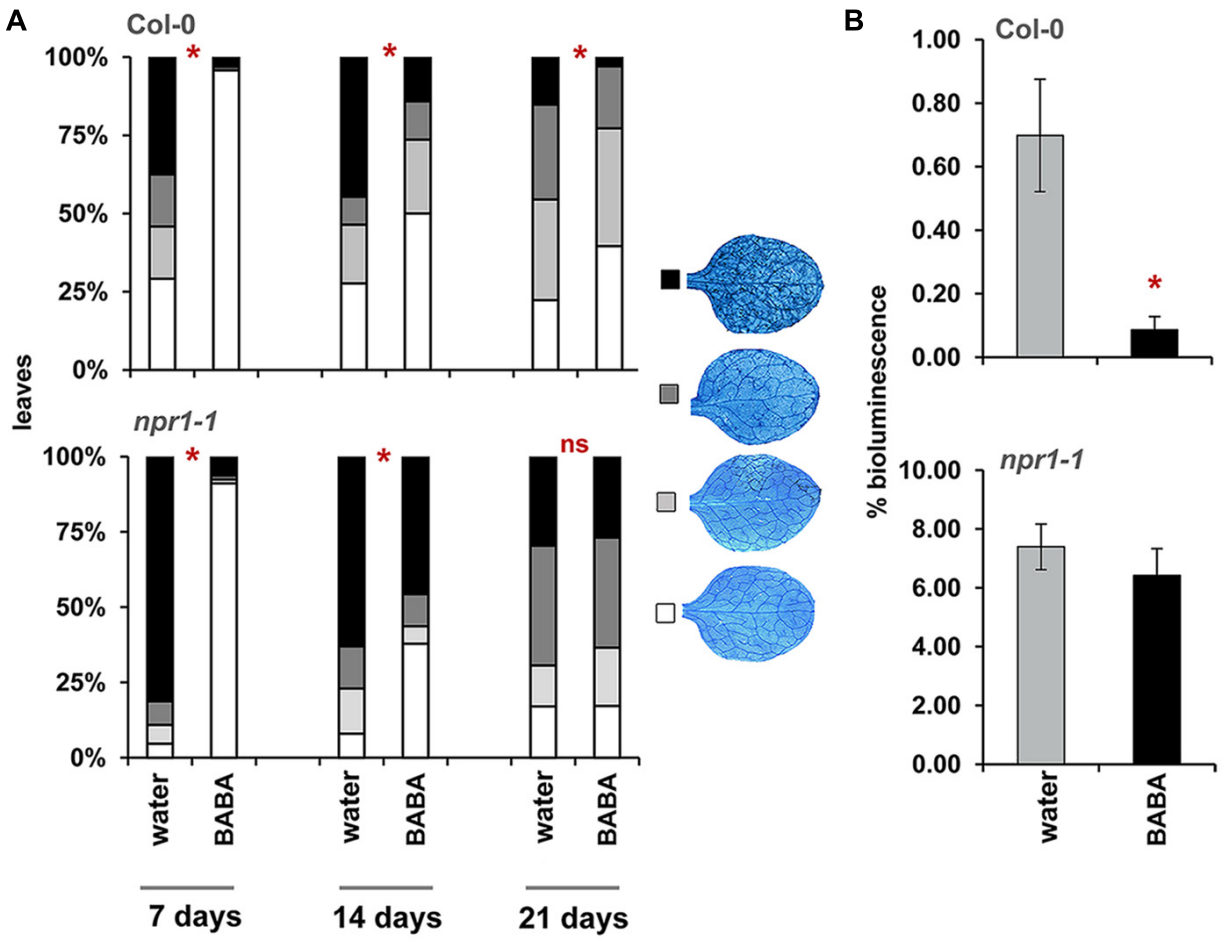
**Durability of BABA-IR in *Arabidopsis***. **(A)** Levels of leaf colonization by *H. arabidopsidis* in wild-type (Col-0) and *npr1-1* plants infected at 7, 14, and 21 days after treatment with 40 mg/L BABA (dat). Shown are % of leaves assigned to four different pathogen colonization classes, based on scoring of 50–100 trypan-blue stained leaves at 6 days after inoculation with 10^5^ spores mL^-1^. Asterisks indicate statistically significant differences between treatments (χ^2^ contingency test; α < 0.05). **(B)** Bacterial colonization by *Pst*-luxCDABE in wild-type (Col-0) and *npr1-1* plants at 28 dat (40 mg/L BABA). Shown are average values (±SEM) of relative bioluminescence per plant at 3 days after inoculation. Asterisk indicates a statistically significant difference between water- and BABA-treated plants (Student’s *t*-test; α < 0.05). ns = no significant.

To determine durability of BABA-IR, plants were infected with *H. arabidopsidis* at 7, 14, and 21 days after BABA application (**Figure 2A**), after which colonization was microscopically analyzed at 6 days after inoculation. Because of age-related resistance against *H. arabidopsidis* at later developmental stages, bioluminescent *Pseudomonas syringae* pv. *tomato* DC3000 (*Pst*-luxCDABE) was used to determine disease resistance at 28 days after induction treatment. BABA-treated plants express nearly complete levels of resistance against *H. arabidopsidis* when inoculated at 7 days after induction treatment, which declined when plants had been inoculated at later time points. Nevertheless, statistically significant levels of induced resistance were still detectable by 21 days after treatment (**Figure 2A**). Moreover, when plants had been infected *Pst*-luxCDABE at 28 days after induction treatment, BABA-treated plants still allowed lower levels of leaf colonization by these bacteria (**Figure 2B**). Hence, BABA-IR declines during the first 2 weeks after treatment, but remains stable during following weeks. Slaughter et al. (2012) demonstrated that plant-endogenous BABA levels at 3 weeks after soil-drench application (40 mg/L) are 10-fold lower than the threshold level required for induced resistance. Accordingly, we conclude that long-lasting resistance by BABA is not due to lingering traces of BABA in the tissue, but rather due to long-lasting physiological changes in the plant.

### LONG-LASTING BABA-IR REQUIRES NPR1

The *npr1-1* mutant is blocked in SA-dependent defense (Cao et al., 1994). Consequently, this mutant is only capable of expressing the SA-independent component of BABA-IR (Zimmerli et al., 2000). To examine which component is responsible for long-lasting disease protection, we measured durability of BABA-IR in *npr1-1* plants. As observed in wild-type plants, *npr1-1* expressed relatively high levels of BABA-IR when the plants had been inoculated at 7 days after priming treatment, which declined as time progressed (**Figure 2A**). However, unlike wild-type plants, BABA-treated *npr1-1* failed to express induced resistance to *H. arabidopsidis* and *Pst*-luxCDABE when inoculated at 21 and 28 days after induction treatment, respectively (**Figure 2**). These results indicate that long-lasting BABA-IR is regulated by the NPR1-dependent pathway.

### LONG-LASTING PRIMING BY BABA IS ASSOCIATED WITH PRIMING OF SA-INDUCIBLE GENES

**FIGURE 3:**
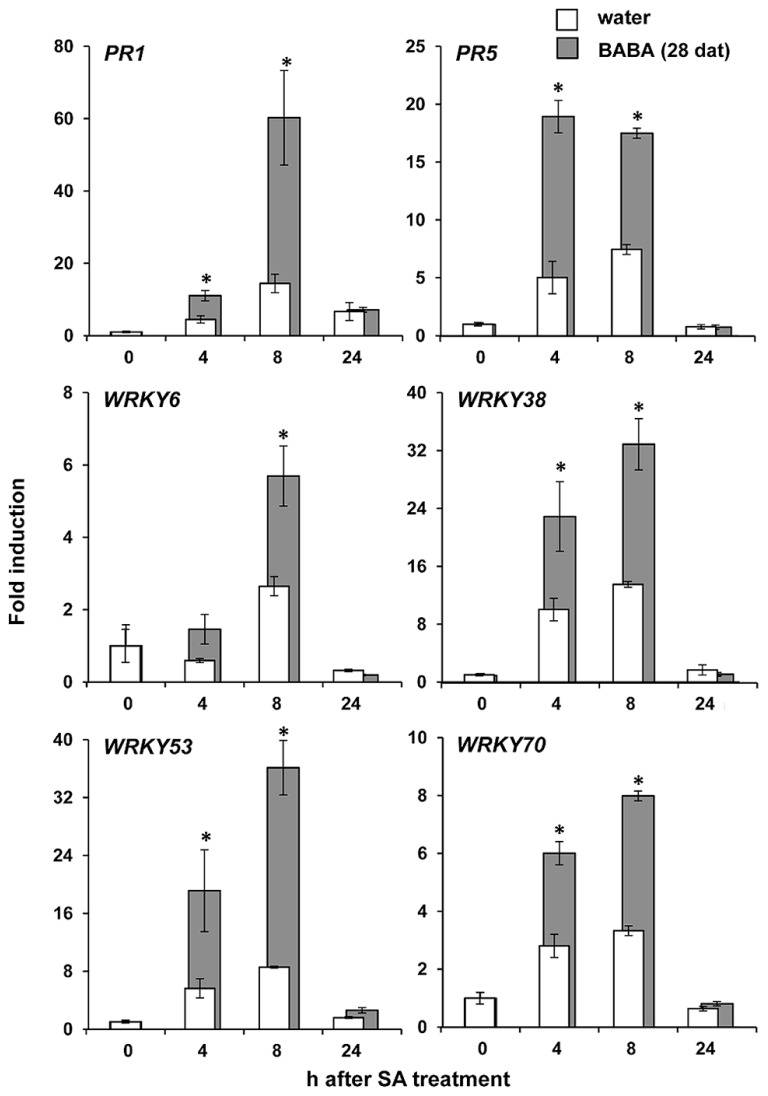
**Long-lasting priming of SA-dependent gene transcription**. Levels of gene transcription were determined by RT-qPCR analysis of shoot tissues from water- and BABA-treated plants at 28 days after treatment (dat) with 40 mg/L BABA, and different hours (h) after spraying of the leaves with 0.5 mM SA. Data represent average fold-change values (±SEM) relative to expression levels before SA application. Asterisks indicate statistically different levels of expression between control- and BABA-treated plants (Student’s *t*-test; α < 0.05; *n* = 3).

NPR1 regulates priming of SA-dependent defense (Kohler et al., 2002; Van der Ent et al., 2009). Since our experiments revealed that NPR1 is necessary for long-lasting resistance by BABA, we investigated whether the resistance is associated with transcriptional priming of SA responsive genes (*PR1, PR5, WRKY70, WRKY6, WRKY53,* and *WRKY38*). At 28 days after BABA application, leaves were sprayed with 0.5 mM SA and harvested at different time-points after treatment for RT-qPCR analysis of defense gene expression. As is shown in **Figure 3**, basal transcription levels of all genes before SA application were similar in BABA- and control-treated plants. However, after application of SA, all genes tested showed faster and stronger transcriptional induction in BABA-treated plants compared to control plants (**Figure 3**). Hence, long-lasting induced resistance is not based on enhanced transcription of SA-dependent defense genes, but rather on a transcriptional priming of these genes.

### SA-DEPENDENT GENE PRIMING IS NOT MARKED BY INCREASED ACETYLATION OF H3K9

**FIGURE 4:**
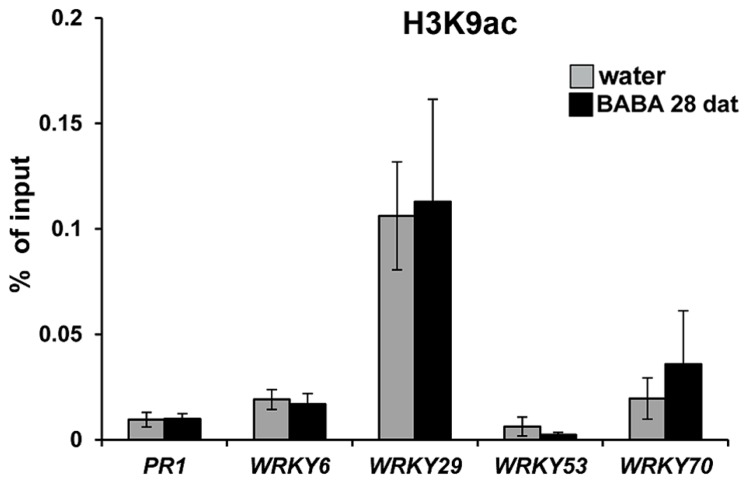
**Long-lasting defense gene priming is not marked by increased H3K9ac**. Levels of H3K9ac at the promoters of the SA-inducible *PR1, WRKY6, WRKY29, WRKY53,* and *WRKY70* genes in water and BABA treated wild-type plants at 28 dat. Chromatin-immunoprecipitation (ChIP) was quantified by qPCR and expressed relative to DNA amounts in chromatin extracts before immunoprecipitation (input). Data represent mean values (±SEM) from three biologically replicated samples.

Chromatin remodeling is an epigenetic mechanism that can provide long-lasting changes in the plant’s transcriptional capacity (Berger, 2007). Post-translational modifications at the lysine residue 9 of histone 3 (H3K9) have been shown to regulate gene transcription (Li et al., 2007). Acetylation of H3K9 correlates with increased transcriptional capacity, whereas methylation of this residue correlates with gene silencing (Zhou et al., 2010). Previously, we demonstrated that transgenerational priming of SA-dependent defense in progeny from diseased *Arabidopsis* is associated with enrichment of H3K9ac at the promoter regions of the primed genes (Luna et al., 2012). This post-translational histone modification has also been associated to short-term defense gene priming after treatment with BTH and BABA (Jaskiewicz et al., 2011; Po-Wen et al., 2013). Based on these observations we assessed levels of H3K9 acetylation in promoters of defense genes displaying long-lasting priming by BABA (**Figure 3**), using similar primer pairs and H3K9ac antibody as described before (Jaskiewicz et al., 2011; Luna et al., 2012). Unexpectedly, these ChIP analyses revealed that BABA did not have long-term impacts on H3K9ac levels at the promoter of *PR1,* nor did it consistently affect H3K9ac levels at promoters of *WRKY* genes (**Figure 4**). These results indicate that H3K9ac is not a *cis*-acting requirement for long-lasting defense gene priming by BABA.

### KYP REGULATES LONG-LASTING DEFENSE GENE PRIMING

**FIGURE 5:**
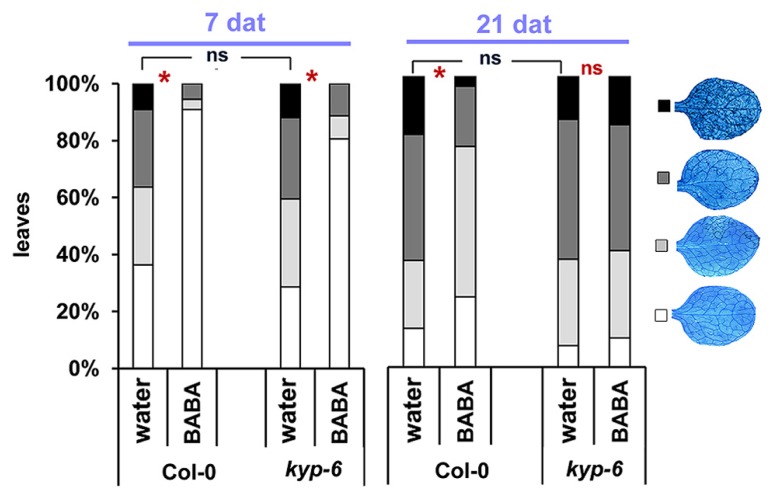
**KYP acts as a positive regulator of long-term maintenance of BABA-IR**. BABA-IR against *H. arabidopsidis* in wild-type and *kyp-6* plants at 7 and 21 days after induction treatment with 40 mg/L BABA (dat). For details see legend of **Figure 2**. Asterisks indicate statistically significant differences between treatments (χ^2^ contingency test; α < 0.05). ns = no significant.

SUVH4/KRYPTONITE (KYP) is a histone methyltransferase that methylates H3K9 residues and its activity results in gene silencing through the interaction with CHROMOMETHYLASE3 (CMT3) DNA methyltransferase (Jackson et al., 2002). Loss of KYP results in decrease H3K9me2 and DNA methylation levels at CpHpG context (Chan et al., 2006). Previously, in an attempt to decipher the epigenetic mechanisms controlling transgenerational immune priming, we identified KYP as a key regulator of this phenomenon (Luna and Ton, 2012). In order to assess the role of this enzyme in long-lasting priming by BABA, we measured levels of BABA-IR against *H. arabidopsidis* at 7 and 21 days after treatment in the *kyp-6* mutant (Jackson et al., 2002, 2004; Chan et al., 2006; Henderson and Jacobsen, 2008). This mutant displayed wild-type levels of basal resistance against *H. arabidopsidis* and showed significant levels of BABA-IR resistance to *H. arabidopsidis* when inoculated at 7 days after induction treatment (**Figure 5**). However, this mutant had lost its ability to express BABA-IR at 21 days after treatment (**Figure 5**), indicating that KYP acts as a positive regulator of long-term maintenance of BABA-IR, possibly by repressing negative regulatory genes of defense priming.

## DISCUSSION

**FIGURE 6:**
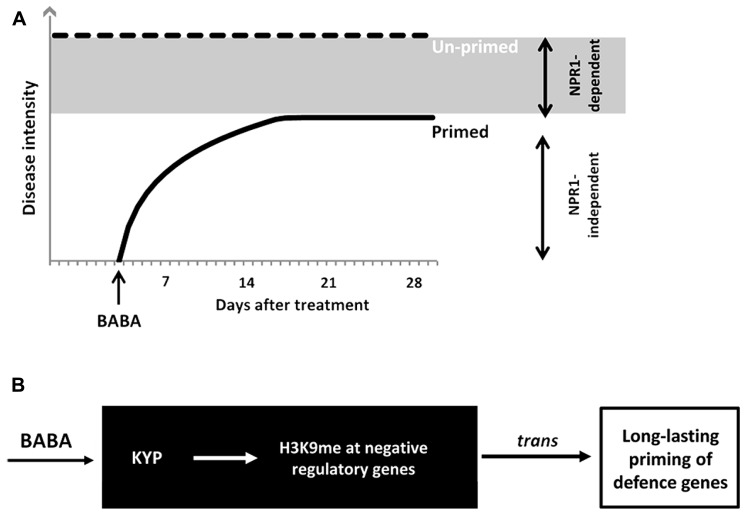
**Model of durability of BABA-IR. (A)** BABA primes SA-inducible defense genes in a NPR1-dependent and -independent manner. BABA-IR lasts up to 28 days in wild-type plants, but only up to 14 days in *npr1-1* plants. Hence, long-lasting disease protection by BABA acts through a NPR1-dependent signaling pathway. **(B)** KYP-mediated control of long-lasting priming by BABA. The *kyp-6* mutant is blocked in long-lasting induced resistance by BABA, but expressed wild-type levels of disease susceptibility. This indicates that KYP stimulates long-lasting defense gene priming by BABA. Since KYP mediates transcriptional silencing through H3K9 methylation and CpHpG DNA methylation, we propose that KYP promotes long-lasting defense priming through silencing of *trans*-acting genes that encode for negative regulators of defense priming.

We have investigated the durability of induced resistance after seedling treatment with the priming agent BABA. The response of plants to this non-protein amino acid mimics different biologically induced resistance responses in plants (Ton et al., 2005; Van der Ent et al., 2009). Consequently, this agent is effective against an exceptionally wide range of diseases (Jakab et al., 2001; Cohen, 2002). BABA primes the plant immune system via at least two independent signaling pathways, which differ in their dependency for NPR1 (Zimmerli et al., 2000). The NPR1-independent pathway primes pathogen-inducible expression of cell wall defense, whereas the NPR1-dependent pathway primes SA-inducible genes (Ton et al., 2005; Van der Ent et al., 2009). In this study, we showed that NPR1-independent resistance by BABA is transient and disappears within 2 weeks after application (**Figure 6A**). On the other hand, NPR1-dependent BABA-IR is long-lasting and remains up to 28 days after treatment (**Figure 6A**). Consistent with the idea that long-lasting BABA-IR involves epigenetic regulation, Slaughter et al. (2012) demonstrated increased defense phenotypes in progeny from isogenic *Arabidopsis* lines upon treatment with BABA. These phenotypes included reduced disease susceptibility to *H. arabidopsidis* and a sensitization to priming treatment by BABA, i.e., plants were “primed to be primed.” Interestingly, transgenerational effects by BABA were largely blocked in the *i*mpaired in BABA-induced sterility1 (*ibs1*) mutant, which had previously been reported to be affected in priming of NPR1-dependent defense by BABA (Ton et al., 2005). Furthermore, we recently demonstrated epigenetic inheritance of NPR1-dependent resistance from *Pst*-luxCDABE-infected *Arabidopsis*, which remained stable up to two generations. We, therefore, conclude that long-lasting NPR1-dependent resistance within the same generation has an epigenetic component, which can, at least partially, be transmitted to following generations.

Previous studies have demonstrated a clear correlation between H3K9 acetylation and transcriptional priming at defense gene promoters (Jaskiewicz et al., 2011; López et al., 2011; Luna et al., 2012; Po-Wen et al., 2013). Using similar gene primers as described earlier (Luna et al., 2012), our ChIP experiments showed that long-lasting priming of SA-inducible defense genes within one generation can occur independently from increased H3K9 acetylation (**Figure 4**). Although we cannot exclude the possibility that flanking promoter regions of these genes did show enhanced H3K9 acetylation, our findings indicate that H3K9 acetylation at the regions analyzed is not a strict requirement for long-lasting priming of SA-inducible genes. The difference between our current H3K9ac results and those reported in progenies from *Pst*-luxCDABE-infected plants (Luna et al., 2012) suggests that the within-generational effects by BABA and transgenerational effects by pathogens are based on different epigenetic mechanisms. This is further supported by the fact that transgenerational resistance by BABA is lost after one stress-free generation (Slaughter et al., 2012), whereas transgenerational resistance by *Pst*-luxCDABE is maintained over 1 stress-free generation (Luna et al., 2012).

Interestingly, the *kyp-6* mutant failed to retain long-lasting induced resistance by BABA (**Figure 5**), suggesting that *KYP* enables long-lasting defense priming by BABA. Although the possibility that the *kyp-6* mutant harbors an independent second mutation disrupting long-lasting BABA-IR cannot be excluded, the phenotype of the *kyp-6* mutant strongly suggests that H3K9 methyltransferase activity by the KYP protein is necessary for long-term maintenance of SA-dependent defense priming in BABA-treated plants. Since KYP-dependent H3K9 di-methylation and associated CpHpG DNA methylation are repressive mechanisms of gene expression (Kass et al., 1997; Zhou et al., 2010), we propose that KYP maintains BABA-IR by silencing repressive regulatory genes of SA-dependent defense genes, thereby priming their responsiveness to pathogen infection (**Figure 6B**). Previously, we proposed that disease-induced repression of RNA-directed DNA methylation is responsible for transmission of defense priming (Luna et al., 2012). Further research is required to resolve the interaction between KYP and components of RNA-directed DNA methylation with regards to long-lasting BABA-IR.

There is no evidence to support that post-translational histone modifications themselves can be transmitted through meiosis in plants. It is, therefore, plausible that transgenerational transmission of BABA-IR is determined by differentially methylated DNA regions (DMRs) that can faithfully be transmitted through meiosis. Indeed, we recently reported that transgenerational resistance in progeny from *Pst*DC30000-infected *Arabidopsis* is most likely transmitted through a reduction in non-CpG DNA methylation (Luna and Ton, 2012; Luna et al., 2012). Interestingly, however, Slaughter et al. (2012) did not detect consistent changes in DNA methylation at priming-responsive defense genes in progenies of BABA-treated plants, indicating that transgenerational priming of defense genes by BABA is regulated by *trans*-acting DMRs. Future research is required to decipher the complex interplay between small RNAs, DNA (hypo)methylation, and post-translational histone modifications (Bond and Baulcombe, 2014).

Safeguarding food security represents an urgent challenge in this century, which is further aggravated by climate change that can render agricultural lands less suitable for crop production. Consequently, there is a pressing need to improve the efficiency of sustainable food production, including intensification of durable crop protection strategies (Royal-Society, 2009). Although usage of modern fungicides poses relatively little direct risks on food safety and soil ecology, repeated applications of fungicides demand considerable energy consumption. Integration of long-lasting induced resistance in existing disease management schemes would allow fewer energy costs to reach similar levels of disease protection. Worrall et al. (2012) recently reported that seed treatment of tomato with BABA provides long-lasting protection against powdery mildew. Considering that some crops are cultivated hydroponically, seedling application of BABA would provide another means of achieving long-lasting induced resistance against disease. Our present study has uncovered chromatin remodeling as an important regulatory mechanism of long-lasting induced resistance. Future research is required to identify the *trans*-acting genes that become targeted by chromatin remodeling after priming treatment, which will help to optimize the efficiency of durable induced resistance in plants.

## Conflict of Interest Statement

The authors declare that the research was conducted in the absence of any commercial or financial relationships that could be construed as a potential conflict of interest.
